# CT features predict tumour invasion of adrenal pheochromocytoma: a retrospective observational study

**DOI:** 10.1186/s12880-025-01827-6

**Published:** 2025-07-14

**Authors:** Sisi Zheng, Rongli Xie, Boke Liu, Jingjing Jiang, Mengsu Zeng, Yuan Ji, Mingliang Wang

**Affiliations:** 1 Department of Radiology, The First Affiliated Hospital of Zhejiang Chinese Medical University (Zhejiang Provincial Hospital of Chinese Medicine), Hangzhou, China; 2 Department of General Surgery, RuiJin Hospital Luwan Branch, Shanghai Jiaotong University, School of Medicine, Shanghai, China; 3 Department of Urology, Ruijin Hospital, Shanghai Jiaotong University School of Medicine, Shanghai, China; 4 Department of Endocrinology, Zhongshan Hospital Fudan University, Shanghai, China; 5 Department of Radiology, Shanghai Geriatric Medical Center, Shanghai, 200032 China; 6 Department of Radiology, Zhongshan Hospital, Fudan University, Shanghai, 200032 China; 7 Department of Pathology, Zhongshan Hospital Fudan University, Shanghai, 200032 China

**Keywords:** Adrenal gland, Pheochromocytoma, Tomography, Computed tomography, Invasiveness

## Abstract

**Objective:**

To investigate the predictive value of the CT features of adrenal pheochromocytoma (PCC) for invasive behaviour.

**Methods:**

From November 2009 to December 2021 at Zhongshan Hospital Affiliated with Fudan University, the clinical and CT data of 148 patients with 163 lesions confirmed by surgery and pathology were retrospectively analysed. The cases were divided into an invasive group and a noninvasive group on the basis of the surgical and pathological results; 27 lesions in 26 patients were classified into the invasive group, whereas 136 lesions in 122 patients were classified into the noninvasive group. Clinical data such as patient age, sex, clinical symptoms, intraoperative blood pressure fluctuations and CT data such as the mean lesion diameter, shape, boundary, calcification, streak sign, and necrosis/cystic degeneration area were compared between the two groups. The density of the solid components of the lesions in the nonenhanced image, arterial phase, and venous phase were measured, and the degree of enhancement, percentage and difference in the degree of enhancement in the different phases were calculated and compared between the two groups statistically.

**Results:**

There were statistically significant differences in the mean diameter, shape, border, streak sign, and tumour blood vessels between the invasive group and the noninvasive group (*P* < 0.05). There was no significant difference in tumour calcification, the proportion or distribution of necrosis/cystic degeneration, or the fluid level in cystic degeneration between the two groups (*P* > 0.05). There were significant differences in the density and degree of enhancement of the solid component of the tumour in the arterial phase and venous phase, as well as the percentage of enhancement in the venous phase, between the two groups (*P* < 0.05). There were no significant differences in the density of the solid components of the tumour on nonenhanced images, the degree of enhancement between the arterial phase and the venous phase, or the percentage of enhancement of the lesions in the arterial phase (*P* > 0.05). The areas under the ROC curves of the arterial-phase density, venous-phase density, arterial-phase enhancement degree, venous-phase enhancement degree, and venous-phase enhancement percentage were 0.618, 0.641, 0.618, 0.639, and 0.635, respectively. The density, enhancement degree and enhancement percentage of the venous phase can be used for discrimination.

**Conclusion:**

Pheochromocytoma with unclear borders, visible tumour blood vessels, and enhancement with the streak sign has predictive value for invasive behaviour, whereas a greater degree of enhancement of the solid component indicates that the tumour has no invasive behaviour.

## Introduction

Pheochromocytoma (PCC) is an accessory ganglion tumour originating in the adrenal medulla [1–3]. In the latest edition of the WHO Classification (4th edition 2017) of Adrenal Tumours, the concept of benign and malignant classification in the original 2014 edition was abandoned, and all PCCs were considered to have metastatic potential [4–6]. PCC usually causes a series of clinical symptoms, such as paroxysmal hypertension, headache, palpitations, and night sweats, through the secretion of catecholamines [7, 8].

Surgical resection is generally recommended in clinical practice, but interference from the tumour during surgery is likely to cause a catecholamine crisis or severe bleeding. Additionally, residual adrenal tissue is likely to cause tumour recurrence. Since puncture biopsy is usually not recommended for preoperative diagnosis, accurate evaluation of the PCC and its potential for metastasis is crucial during the perioperative period [9–15]. With the advent of the new classification system, CT parameters for the differentiation of benign from malignant PCCs may no longer be applicable, necessitating further research. This study aimed to explore the value of CT signs in predicting the invasive behaviour of PCCs by analysing the clinical and CT imaging data of PCC patients.

## Materials and methods

### Patient characteristics

All enrolled patients underwent surgical resection and had pathologically confirmed adrenal PCC at Zhongshan Hospital of Fudan University from November 2009 to December 2021. The inclusion criteria were as follows: surgical pathology confirmed adrenal PCC; patient underwent preoperative CT with contrast two weeks before surgery; images from the noncontrast scan and arterial and venous phases were complete; no concomitant primary endocrine tumours or malignant tumours in the adrenal region were present; and the clinical data were complete. The exclusion criteria were as follows: recurrent or metastatic lesions after adrenal PCC surgery, treatment before CT examination, and incomplete images at the lesion level or an incomplete scanning period.

This retrospective study was approved by the Ethics Committee of Zhongshan Hospital of Fudan University. All the procedures were implemented in accordance with the principles of the Declaration of Helsinki. Since this was a retrospective study and anonymized data were evaluated, patient consent was waived by our institutional ethics committee.

### CT scan

A Somatom Definition AS 64-slice spiral CT scanner (Siemens Healthineers, Erlangen, Germany) or a 40-slice or a 128-layer CT scanner (United Imaging Health care Technology Co., Ltd., China) was used to acquire the images. The scanning parameters were as follows: tube voltage, 120 kVp; automatic tube current, 210–250 mAs; reconstruction layer thickness, 2.5–5 mm; and layer spacing, 2.5–5 mm. All patients underwent double-phase enhanced CT scanning after conventional noncontrast CT scanning, and iopromide (iodine concentration of 300 mg/ml; Bayer-Schering, German) was injected through the anterior elbow vein at a flow rate of 2–3 mL/s with a double-barrel high-pressure syringe. The arterial phase images were collected by a contrast agent tracking trigger with a trigger threshold of 100 HU, and the venous phase images were collected 70 ~ 80 s after the injection.

### CT scan analysis

**Fig. 1:**
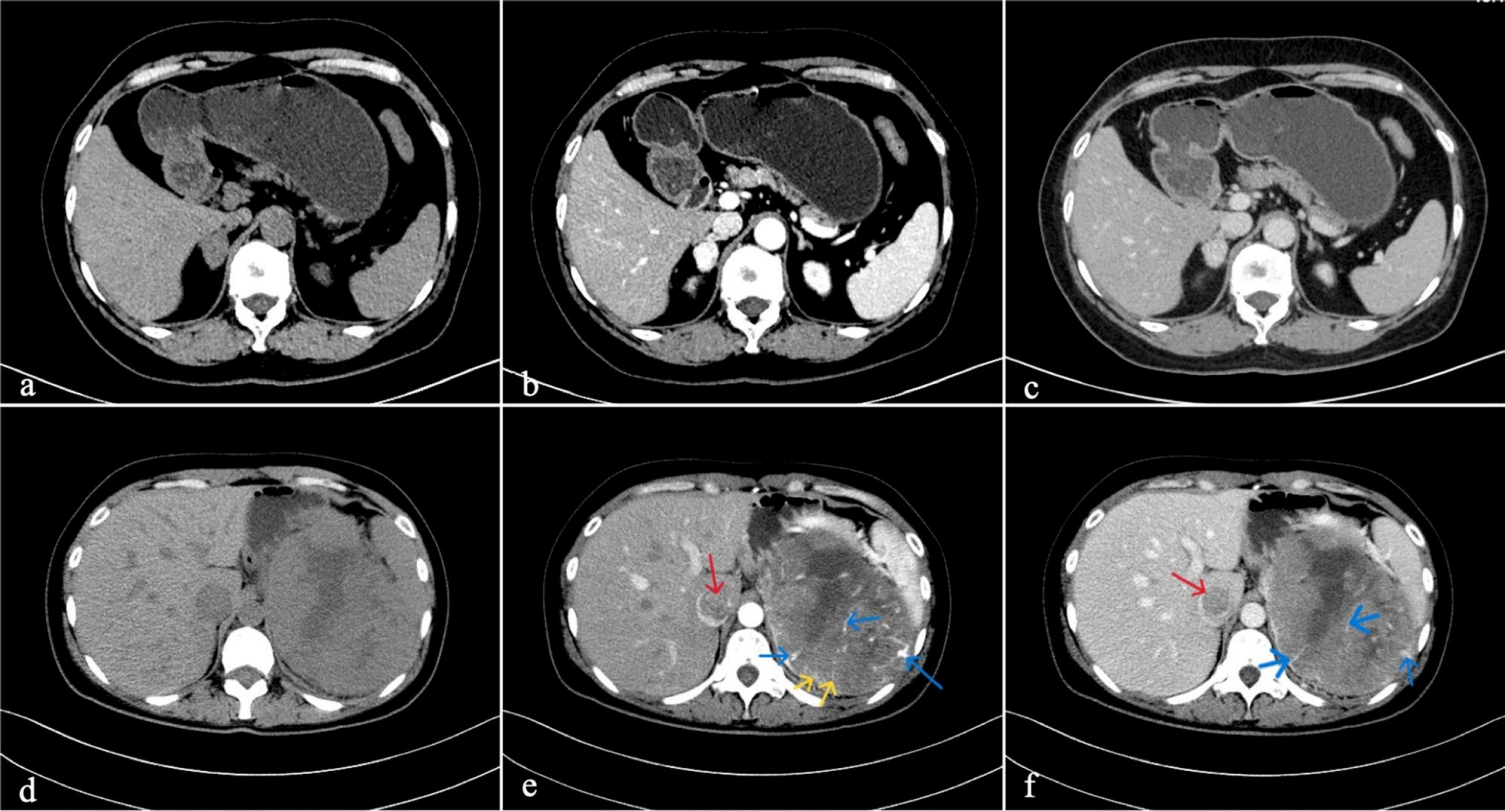
**a**-**c**. A patient with a noninvasive PCC in the right adrenal gland; the PCC had a quasicircular shape, clear boundary, and uniform density (44.8 HU) with uniform noncontrast. Obvious uneven enhancement was observed in the arterial phase, the CT value of the most obvious area of tumour solid component enhancement was approximately 168.0 HU, and the CT value of the venous phase decreased to 135.3 HU. Figure 1**d** and **f**. A patient with an invasive PCC in the left adrenal gland the PCC had a lobulated shape, unclear boundaries, and uneven density. Moreover, the streak sign (yellow arrow) and tumour vascular shadow (blue arrow) were observed in the posterior arterial phase of enhancement, and thrombus can be seen in the inferior vena cava (red arrow), and pathology suggested that the lesion had invaded the capsule

All the CT images were analysed by two independent radiologists through picture archiving and communication systems (PACSs). The qualitative and quantitative indexes of lesions were analyzed in qualitative indicators and quantitative indicators. When the qualitative indicators are inconsistent, a third senior doctor will participate in the discussion. The quantitative indicators take the average value. The qualitative indicators were as follows: shape (circular, oval, lobulated and irregular), proportion of necrosis/cystic degeneration (< 30%, 30–70%, > 70%), site of necrosis/cystic degeneration (none, centre, eccentric), calcification or not, boundaries (clear, unclear), tumour vessels (internal or marginal vascular shadows), and the streak sign (arterial solid component, multiple streak-like enhancement manifestations) (shown in Fig. 1).

The quantitative indicators used were size, CT values and the selection of the region of interest (ROI). The size index was defined as follows: average diameter = (long diameter + short diameter) ÷ 2, where the long diameter is the maximum diameter of the maximum cross section of the tumour, and the short diameter is the maximum diameter perpendicular to the long diameter. The measurement results are the average of three measurements.

The CT values included noncontrast scans, arterial phase images and venous phase images. The degree of enhancement in the arterial phase/venous phase = CT values of (arterial phase/venous phase - noncontrast period), the difference between the arterial phase and the venous phase in the degree of enhancement = CT values of (arterial phase - venous phase), and the percentage of lesion enhancement in the arterial phase/venous phase = CT values of ((arterial phase/venous phase-noncontrast period) ÷ noncontrast × 100%).

The ROI was placed where the enhancement of the arterial phase of the lesion was most prominent, avoiding necrotic, cystic degeneration, calcification and vascular areas.

### Statistical analysis

Statistical analysis was performed using SPSS version 26.0. Categorical variables were compared with the chi-square test or Fisher’s exact test. Tests for the assumptions of normality distribution and variance homogeneity were performed. Continuous variables are expressed as the means ± SDs for normally distributed data. Non-normal distributed data are represented by the median (upper and lower quartiles). Continuous variables were compared via two-tailed Student’s t tests for normally distributed data and the Mann‒Whitney U test for nonnormally distributed data. The receiver operating characteristic (ROC) curve was used to calculate its sensitivity, specificity, area under the curve (AUC), threshold and 95% confidence interval (CI) to evaluate the diagnostic efficacy of tumour indices on the aggressive behaviour of PCC. P values < 0.05 were considered statistically significant.

## Results

### Patient characteristics

In this study, a total of 150 patients were enrolled. Among these 150 patients, 1 patient with incomplete images in the arterial phase and 1 patient who underwent reoperation after recurrence were excluded, and 148 patients with 163 lesions (including 1 patient with 2 lesions of the unilateral adrenal gland, 8 patients with bilateral double lesions, 1 patient with 3 lesions, and 1 patient with 5 lesions) were analysed after enrolment. There were 72 men and 76 women, ranging from 17 to 80 years old, with an average age of 47.9 ± 14.3 years. The main clinical symptoms included headache, palpitations, and night sweats in 63 patients; persistent hypertension in 93 patients; paroxysmal hypertension in 36 patients; and abdominal pain or discomfort in 14 patients. Only 38 cases were found incidentally by physical examination, with no clinical symptoms.

In this study, PCCs with one or more pathologically visible masses invading the adrenal envelope, surrounding adipose tissue, nerve tract, venous wall, or vascular thrombus were defined as aggressive. The pathological absence of any of the above manifestations was defined as noninvasive. In this study, a total of 26 patients with 27 lesions in the invasive group and 122 patients with 136 lesions in the noninvasive group were included. The clinical data, including sex, age, blood pressure, clinical symptoms and intraoperative blood pressure fluctuations, were compared between the invasive group and the noninvasive group. Among the 26 patients (27 lesions in total) in the invasive group, 11 patients had capsular invasion, 3 had vascular tumour thrombus, and 12 had simultaneous invasion at 2 or more sites. There were no significant differences in age, sex, clinical symptoms, or intraoperative blood pressure fluctuations between the invasive group and the noninvasive group (*P* > 0.05) (Table 1).

**Table 1:** Comparison of clinical data of PCCs in the invasion group and non-invasion group

Group	invasion group	non-invasion group	Statistical value	*P*
Cases	26	122		
Age	46.1 ± 13.5	48.3 ± 14.5	0.726	0.469
Gender				
Male	14	58	0.341	0.559
Female	12	64		
Clinical symptoms				
Yes	20	90	0.112	0.738
No	6	32		
Intraoperative fluctuations in blood pressure				
Yes	16	66	0.480^b^	0.488
No	10	56		

### CT morphological manifestations

The analysis revealed statistically significant differences in the mean diameter, shape, boundary, streak sign and tumour vasculature between the invasive group and the noninvasive group (*P* < 0.05). The diameter of the tumours in the invasive group was greater than that in the noninvasive group, and lobulation or irregular morphology, unclear lesion boundaries and streak signs were more common in the invasive group than in the noninvasive group. However, there were no significant differences in tumour location, calcification, proportion or distribution of necrotic/cystic degeneration changes, or fluid level between the two groups (*P* > 0.05) (shown in Table 2; Fig. 1).

**Table 2:** Comparison of the qualitative indicators in CT imagine between the invasion group and the non-invasion group

Group	invasion group	non-invasion group	Statistical value	*P*
Number	27	136		
Diameter(mm)	47.9(31.5, 62.5)	37.6(24.5, 46.9)	-2.051	0.040
Shape				
Circular / oval	17	111	4.649	0.031
Lobulated / irregular	10	25		
Proportion of NCD				
< 30%	24	101	2.643	0.221
30 − 70%	0	10		
> 70%	3	25		
Site of NCD				
None	19	88	2.118	0.347
Center	0	10		
Eccentric	8	38		
Calcify				
Yes	3	8	0.324	0.569
No	24	128		
Liquid plane				
Yes	2	18	0.272	0.602
No	25	118		
Boundaries				
Clear	22	130	0.205	0.008
Un-clear	5	6		
Streak sign				
Yes	13	27	9.740	0.002
No	14	109		
Tumor vessels				
Yes	10	25	4.649	0.031
No	17	111		

NCD, necrosis/cystic degeneration

### Characteristics of enhancement

There were statistically significant differences in the arterial phase density, venous phase density, arterial phase enhancement, venous phase enhancement and percentage of venous phase enhancement between the two groups (*P* < 0.05), and the degree of enhancement in the arterial phase/venous phase and the percentage of venous phase lesion enhancement in the invasive group were lower than those in the noninvasive group. However, there were no significant differences in the density of solid tumour components, the degree of enhancement between the venous and arterial phases, or the percentage of lesions in the arterial phase (*P* > 0.05) (Table 3).

**Table 3:** Comparison of the quantitative indicators in CT imagine between the invasion group and the non-invasion group

Group	invasion group	non-invasion group	Statistical value	*P*
Number	27	136		
CT value in NCT	40.2 ± 6.6	39.1 ± 5.9	-0.924	0.357
CT value in AP	108.8 ± 25.9	121.6 ± 33.9	2.217	0.032
CT value in VP	87.7 ± 14.2	96.8 ± 19.6	2.290	0.023
Degree of APE	68.5 ± 28.0	82.7 ± 35.7	2.292	0.027
Degree of VPE	47.5 ± 15.0	57.5 ± 21.1	2.344	0.020
Difference between APE and VPE	21.1 ± 14.6	24.8 ± 22.8	54.983	0.280
The percentage of APE	2.2(1.43, 3.01)	1.8(1.16, 2.42)	-1.797	0.072
The percentage of VPE	1.2(0.86, 1.53)	1.5(1.01, 1.94)	-2.214	0.027

NCT, Noncontrast CT; AP, arterial phase; APE, arterial phase enhancement; VP, venous phase. VPE, venous phase enhancement

**Fig. 2:**
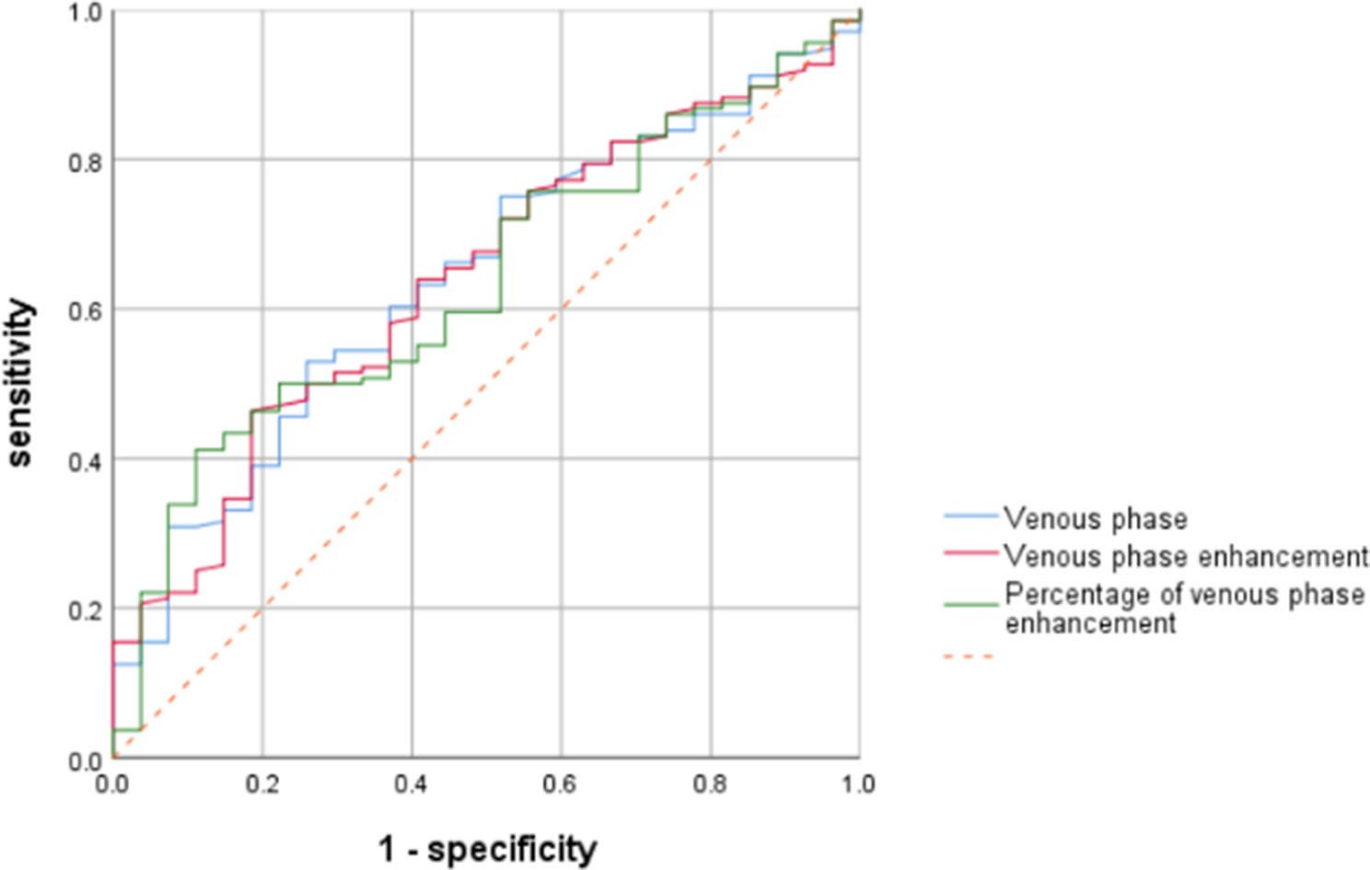
ROC curves of CT scan parameters in the venous phase for predicting the aggressiveness of PCCS. The AUC values of venous phase density, degree of venous phase enhancement and percentage of venous phase enhancement were 0.641, 0.639 and 0.635, respectively

The AUC values of the ROC curves for predicting the aggressive behaviour of PCCs were 0.618 (95% CI 0.517–0.720, *P* = 0.053), 0.641 (95% CI95% CI 0.536–0.747, *P* = 0.020), 0.618 (95% CI 0.515–0.722, *P* = 0.052), 0.618 (95% CI 0.515–0.722, *P* = 0.052), 0.639 (95% CI 0.534–0.745, *P* = 0.022), and 0.635 (95% CI 0.531–0.739, *P* = 0.027). The results revealed that venous phase density, venous phase enhancement and the percentage of venous phase enhancement had discriminative power for predicting the aggressive behaviour of PCCs, with optimal cut-off values of 94.7 HU, 58.3 HU and 1.67; sensitivities of 0.741, 0.815 and 0.889; and specificities of 0.529, 0.463 and 0.412, respectively (as shown in Table 4; Fig. 2).

## Discussion

Pheochromocytoma (PCC) is an accessory ganglion tumor originating in the adrenal medulla [1–3], and the accurate judgment of PCC and its metastasis potential is crucial during perioperative period [16–20]. With the advent of the new classification system, CT parameters for the differentiation of benign from malignant PCCs may no longer be applicable, necessitating further research. This study aimed to explore the value of CT signs in predicting invasive behavior of PCCs and demenstrated that, PCCs with unclear borders, visible tumor blood vessels, enhancement with streak sign has predictive value in invasive behavior, while a higher degree of enhancement of the solid component indicates that the tumor has no invasive behavior.

There are no uniform diagnostic criteria for the histoclinicopathologic classification of adrenal PCC. It is widely accepted that all adrenal PCCs are potentially aggressive and metastatic [3, 21], and accordingly, “metastatic and nonmetastatic pheochromocytoma” is used instead of “benign and malignant pheochromocytoma” in the World Health Organization (WHO) (2017) endocrine tumour classification [22].

In accordance with the latest edition of the WHO New Classification of Adrenal Tumours, this study divided adrenal PCCs into invasive groups and noninvasive groups. The invasive group accounted for approximately 17.6%, which was consistent with the proportion of metastatic PCCs and paragangliomas reported in the literature of approximately 20% [3, 23–25]. Additionally, there was no statistically significant difference in clinical manifestations or intraoperative blood pressure fluctuations between the two groups (*P* > 0.05), which was consistent with the study by Reisch N et al. [26]. Moreover, there were slightly more male patients than female patients in the invasive group, and the opposite was true in the noninvasive group. Approximately three-quarters of the patients in both groups had clinical symptoms (including persistent hypertension, paroxysmal hypertension, headache, palpitations, night sweats, abdominal pain or abdominal discomfort), and there were slightly more patients with intraoperative blood pressure fluctuations than those without.

In terms of CT scan performance, there was no statistically significant difference in the proportion or distribution of necrotic/cystic degeneration changes, calcified lesions (or not), or fluid level in the cystic area (or not) between the two groups (*P* > 0.05). Although there were statistically significant differences in the arterial phase density, degree of arterial phase enhancement and percentage of enhancement, the difference had no discriminatory effect on whether the PCC had aggressive behaviour. It has been suggested that when the diameter of tumours is larger than 5 cm, the risk of metastasis is increased, the overall survival is shorter [7], and thus tumour size can be used as an independent prognostic criterion. The TNM stage of the AJCC also uses a maximum diameter of 5 cm as the basis for staging [21, 27]. In this study, we demonstrated that the average diameter in the invasive group was greater than that in the noninvasive group and that the average diameter in the invasive group was approximately 47.9 mm, which further supported these findings.

Furthermore, we found that a greater percentage of lesions in the invasive group had a lobulated and irregular morphology and unclear lesion boundaries, which may be related to the metastatic behaviour of tumour cells according to pathology, and metastatic tumours are generally more heterogeneous and aggressive [28]. The streak sign and tumour blood vessels were more common in the invasive group than in the invasive group, possibly because invasive behaviour requires a greater blood supply. The streak sign was an abnormally significant streak enhancement of the tumour parenchyma, and the degree of enhancement was close to that of tumour blood vessels; whether there is a pathophysiological basis for this association is worthy of further investigation. Except for the above findings, the noninvasive group had a greater degree of enhancement of the solid components. It was speculated that invasive and noninvasive PCCs are both blood-rich tumours, and when they tend towards malignant biological behaviour, the microenvironment changes, which affects the degree of strengthening of the solid components of the tumour.

The results of the ROC curve analysis in this study revealed that venous phase density, venous phase enhancement degree and the percentage of venous phase enhancement had high sensitivity in discriminating whether PCCs had aggressive behaviour. Since accurate judgement of PCC and its potential for metastasis is crucial during the perioperative period, the aforementioned findings are expected to have potential application value in the clinic and improve the outcomes of PCC patients.

While the current study focused on characterizing the adrenal PCC by contrast-enhanced CT, but the role of radiolabeled somatostatin receptor analogs in the comprehensive evaluation of PCCs/PGLs should also be recognized. Radiolabeled somatostatin analogs, such as 68Ga-DOTA, 18 F-FDOPA and 123I/131I-MIBG for PET/CT, provide high sensitivity and specificity for detecting SSTR-expressing PCCs/PGLs [29–36]. These nuclide examination results focus more on the discovery of metastatic foci, when the metastasis is the ultimate manifestation of invasive behavior [37, 38]. Although these examinations have advantages in evaluating the lesions and metastases of PCC, their resolution for the primary tumor is limited and it is difficult to assess the fat infiltration and vascular invasion of the tumor. CT features with functional imaging findings could further refine risk stratification and management strategies for PCCs/PGLs.

### Limitation

There are several limitations to this study. Firstly, this study is a retrospective analysis of clinical and CT data, and the CT machines and scanning parameters used are not completely unified, which has a certain impact on the results. Also, a thickness of 5 mm can hinder an accurate quantitative measurement of the lesion, especially if the lesion is small. Further prospective research with larger numbers of patients and unified parameters from multiple centers are necessary to validate the result. Secondly, in terms of observing the boundary and cystic components of the tumour, the soft tissue resolution of CT is not as high as that of MRI, and the comparative imaging performance of CT combined with MRI is worthy of further study. Finally, dut to manually marking by clinical physicians, the AUC values indicated modest discriminatory power. In the future, we will use software for deep learning modeling, hoping to achieve a diagnosis model with a higher AUC.

## Conclusion

In summary, there were differences between aggressive and noninvasive PCCs in terms of the mean diameter, shape, border, streak sign, tumour vasculature and solid component of the tumour, arterial phase density, venous phase density, arterial phase enhancement, venous phase enhancement, and percentage of venous phase enhancement. CT signals such as unclear tumour boundaries, visible tumour blood vessels, and enhancement with streaks indicate aggressive behaviour, whereas a higher degree of enhancement of solid tumour components indicates noninvasive behaviour.

**Table 4:** Comparison of ROC valule in CT imagine between the invasion group and the non-invasion group

Variables	AUC value	95% CI		*P*
		Lower limit	Upper limit	
AP	0.618	0.517	0.720	0.053
VP	0.641	0.536	0.747	0.020
APE	0.618	0.515	0.722	0.052
VPE	0.639	0.534	0.745	0.022
Percentage of APE	0.610	0.502	0.717	0.072
Percentage of VPE	0.635	0.531	0.739	0.027

AP, arterial phase; APE, arterial phase enhancement; VP, venous phase. VPE, venous phase enhancement

## Data Availability

The datasets analysed during the current study available from the corresponding author on reasonable request.
